# Usability assessment of a mobile app for patients with peripherally inserted central catheters

**DOI:** 10.1590/1518-8345.5817.3666

**Published:** 2023-01-06

**Authors:** Aline Nair Biaggio Mota, Ruth Natalia Teresa Turrini

**Affiliations:** 1 Universidade de São Paulo, Escola de Enfermagem, São Paulo, SP, Brazil.

**Keywords:** Central Venous Catheters, Information Technology, Telenursing, Telemedicine, Biomedical Technology Assessment, Nursing Informatics, Cateteres Venosos Centrais, Tecnologia da Informação, Telenfermagem, Telemedicina, Avaliação de Tecnologias de Saúde, Informática em Enfermagem, Catéteres Venosos Centrales, Tecnología de la Información, Teleenfermería, Telemedicina, Evaluación de la Tecnología Biomédica, Informática Aplicada a la Enfermería

## Abstract

**Objective:**

to evaluate usability of the *Meu PICC* (My PICC) app for follow-up of outpatients using peripherally inserted central catheters through the validated System Usability Scale instrument.

**Method:**

a cross-sectional study that applied the System Usability Scale to 30 patients using peripherally inserted central catheters, ten nurses and eight Information and Communication Technology professionals to assess usability of the app.

**Results:**

a statistical difference was observed between age and usability (p=0.006), as well as a negative correlation between app use time and usability (p=0.002). As *per* the System Usability Scale adjectival classification, 40.0% and 33.3% of the patients considered the app as the best possible to be imagined and as excellent, respectively. In relation to the nurses, 70.0% considered the app as the best possible to be imagined and 20.0% as excellent; of the Information and Communication Technology professionals, 50.0% considered the app as the best possible to be imagined and the other 50.0%, as excellent.

**Conclusion:**

the usability assessment showed that patients, nurses and ICT professionals considered the app useful for monitoring patients using PICCs and evaluated it as appropriate, evaluating it as the best possible to be imagined or as excellent. These results corroborate use of the *Meu PICC* app in the monitoring of outpatient use of PICCs.

## Resumo

**Objetivo:**

avaliar a usabilidade do aplicativo *Meu PICC* para o acompanhamento extra-hospitalar de pacientes em uso de cateter central de inserção periférica por meio do instrumento validado *System Usability Scale*.

**Método:**

estudo transversal que aplicou o *System Usability Scale* a 30 pacientes em uso de cateter central de inserção periférica, dez enfermeiros e oito profissionais de tecnologia da informação para avaliar a usabilidade do aplicativo.

**Resultados:**

observou-se diferença estatística entre idade e usabilidade (p=0,006), bem como correlação negativa entre o tempo de utilização do aplicativo e usabilidade (p=0,002). Pela classificação adjetiva do *System Usability Scale,* 40,0% dos pacientes consideraram o aplicativo como o melhor imaginável e 33,3% como excelente. Com relação aos enfermeiros, 70,0% consideraram o aplicativo como o melhor imaginável e 20,0% como excelente; dos profissionais de tecnologia da informação, 50,0% consideraram o aplicativo como o melhor imaginável e 50,0% excelente.

**Conclusão:**

a avaliação de usabilidade mostrou que pacientes, enfermeiros e profissionais de TIC consideraram o aplicativo útil para o acompanhamento de pacientes em uso de PICC e o avaliaram como adequado, atribuindo-lhe a qualificação de melhor imaginável ou excelente. Estes resultados corroboram para o uso do aplicativo *Meu PICC* no acompanhamento de pacientes em uso extra-hospitalar de PICC.

## Resumen

**Objetivo:**

evaluar la usabilidad de la aplicación *Meu PICC* para el seguimiento extrahospitalario de pacientes que usan catéter central de inserción periférica por medio del instrumento validado *System Usability Scale*.

**Método:**

estudio transversal que aplicó el *System Usability Scale* a 30 pacientes que usan catéter central de inserción periférica, diez enfermeros y ocho profesionales en tecnología de la información para evaluar la usabilidad de la aplicación.

**Resultados:**

hubo diferencia estadística entre la edad y la usabilidad (p=0,006), así como una correlación negativa entre el tiempo de uso de la aplicación y la usabilidad (p=0,002). Según la escala de clasificación de adjetivos del *System Usability Scale*, el 40,0% de los pacientes consideró que la aplicación era mejor imaginable y el 33,3% excelente. En cuanto a las enfermeras, el 70,0% consideró que la aplicación era mejor imaginable y el 20,0% excelente; de los profesionales en tecnologías de la información, el 50,0% consideró que la aplicación era mejor imaginable y el 50,0% excelente.

**Conclusión:**

la evaluación de usabilidad determinó que los pacientes, enfermeros y profesionales en TIC consideraron que la aplicación era útil para el seguimiento de pacientes que utilizan PICC y la calificaron como adecuada, otorgándole la calificación de mejor imaginable o excelente. Estos resultados confirman el uso de la aplicación *Meu PICC* en el seguimiento de pacientes que usan PICC fuera del hospital.

**Highlights**

(1) 40.0% of the patients considered the app as the best one possible to be imagined.

(2) A statistical difference was observed between age and usability (p=0.006).

(3) A negative correlation was observed between app use time and usability.

(4) Daily access to the Internet was mentioned by 29 (96.7%) patients.

## Introduction

Use of Peripherally Inserted Central Catheters (PICCs) has been expanded to different care areas due to the lower risk of complications during insertion, when compared to centrally inserted central catheters, to good durability for medium to long-term Infusion Therapy (IT) and to the possibility of outpatient treatment, with an improvement in the patient’s quality of life and optimization of hospital resources and beds^1^. 

For outpatient IT, the eligible patients are those clinically stable, with good adherence to the treatment, and conditions in phases with low risk of complications. The main indications are treatments with antimicrobials, chemotherapy drugs, hydration, parenteral nutrition and analgesics^2^ ^-^ ^4^. In order to ensure IT continuity, it is important to incessantly seek to mitigate the complications. In the outpatient environment, these measures should involve education of patients and caregivers regarding purpose of the treatment, type and duration of the therapy prescribed, risks and benefits involved, possible adverse effects, potential complications of the intravenous device, care plan, and information on how to access the health service^2^ ^-^ ^4^. 

Education in health is related to “health literacy” a term used in the literature to designate the extent to which individuals have the ability to find, understand and use diverse information and services to communicate health-related decisions and actions. “Health literacy” is considered a complex phenomenon that involves individuals, families, communities and systems. Its concept covers the materials developed for patient education, the environments involved, and the challenges specifically associated with health conditions and treatment and prevention measures. In addition to that, it involves a high number of skills such as reading, understanding and analyzing information, following instructions, performing calculations, decoding symbols and interpreting graphs and diagrams^5^.

Patients with a low level of “health literacy” are more likely to have unfavorable health outcomes, as it is associated with lower adoption of preventive behaviors, lower adherence to treatments and more frequent hospitalizations^6^. In addition to “health literacy”, the Infusion Nurses Society (INS) recommends that factors such as age, degree of cognitive development, access to resources and technologies, the patient’s preferences and possible cultural influences are also considered during development of each patient’s health education strategy^4^. 

To enable education in health and outpatient follow-up, the use of Information and Communication Technology (ICT) by health professionals has expanded worldwide. As using these technologies promotes the dialogic and participatory education in health advocated by Paulo Freire, a process of awareness raising of patients beyond the hospital environment is initiated, through the co-construction of knowledge from their own contexts and demands at home^7^. Thus, the number of studies that address different types of interactions with patients with the objective of guiding, monitoring and encouraging adherence to the treatment of chronic, contagious and psychiatric diseases has increased^8^ ^-^ ^10^.

There is still a lack of studies examining the results related to central vascular access devices outpatient care and development of complications^11^. To date, a meta-analysis of 36 Chinese studies with 2,623 controls and 2,662 patients who used the WeChat app for monitoring outpatients with PICC showed that the group that underwent follow-up through the app presented a lower risk of PICC-related complications with an odds ratio (OR) of 0.23 (p<0.00001), better self-care ability with a mean difference of 36.4 (p<0.00001), greater dependence on PICC maintenance with an OR of 4.27 (p<0.00001) and greater patient satisfaction with an OR of 6.20 (p<0.00001)^12^.

At the hospital institution of this study, at the time of discharge and according to the institutional protocol, the patients receive guidelines for safe maintenance of the PICCs in the outpatient environment, in order to preserve integrity of the devices and identify complications. As they are central vascular access devices, with rapid capacity for systemic deterioration in case of complications and demand for specific care, it was deemed necessary to develop a smartphone app to allow patients to access these guidelines and ease communication with the nurse of the institution at any time and place.

Prior to this study, the PICC patient discharge protocol in force at the research institution was reviewed in accordance with the standardized practices for the use of IT^4^, the infection control measures related to invasive venous devices set forth by the International Nosocomial Infection Control Consortium (INICC)^13^ and the Brazilian National Health Surveillance Agency^14^, and the guidelines by the Hospital Infection Control Committee (*Comissão de Controle de Infecção Hospitalar*, CCIH) and the Infusion Therapy Group of the institution itself, subsequently validated by experts and patients using PICCs. After obtaining this updated content, the *Meu PICC* app for smartphones was developed for the monitoring of outpatients in use of PICCs. 

It is important that the development of apps goes through a usability assessment, as it is considered a product quality attribute that involves five components, namely: ease of learning, efficiency, ease of memorization, minimization of errors, and satisfaction^15^. Such components can be explored in greater or lesser depth according to the objective of the app and to the assessment instrument used.

Thus, this study aimed at assessing usability of the *Meu PICC* smartphone app for the follow-up of outpatients using peripherally inserted central catheters through the System Usability Scale (SUS).

## Method

### Study type and period

A cross-sectional study was conducted between September 2020 and January 2021.

### Study locus

The study was carried out in inpatient units and in the day hospital of a tertiary-level health care teaching hospital specialized in Cardiopneumology from the city of São Paulo, SP, Brazil, which meets a demand of approximately 13,000 hospitalizations per year. The hospital has 535 beds, 157 of which are in the Intensive Care Unit. PICC insertion is in charge of duly certified and qualified nurses and follows the institutional protocol for insertion, maintenance and removal. Administration of antimicrobials and antivirals represents the most commonly used type of IT via PICCs. 

### Population

The study had three different groups of participants to enable usability assessment of the app, from different points of view: patients with PICCs for IT; nurses and ICT professionals from the same institution.

### Inclusion criteria

The patients should use PICCs, have the Public Health System as a provider, be over 18 years of age, and state mastery in the use of a smartphone with a camera and Internet access. The nurses of the institution should work or have previous experience in the care of outpatients in use of PICCs in the last five years. The ICT professionals should have Complete Higher Education in the areas of Systems Analysis and Development.

### Exclusion criteria

The patients excluded were those with cognitive and spatial-temporal alterations or psychiatric diseases without a caregiver or not in due clinical conditions for smartphone manipulation, according to data available in the patient’s Electronic Health Record (EHR).

### Sample

The sample was intentional and non-probabilistic and recruitment occurred according to the availability of the participants present in the institution during the data collection period and the COVID-19 pandemic.

One of the most renown experts in software usability considers a sample of five users as enough to identify usability problems^15^, in contrast to NBR ISO/IEC 14598-6^16^, which suggests a minimum of eight participants for consistent results. Thus, for this study, we included 30 patients using PICCs at the time of data collection, ten nurses with experience in the care of outpatients with PICCs and eight ICT professionals.

### Study variables

To assess usability of the *Meu PICC* app, the System Usability Scale instrument validated for Portuguese^17^ was used, consisting of 10 items and five-point Likert-type answers that ranged from “I totally disagree” to “I totally agree”. The structure of the questions alternates between positive aspects (odd questions) and negative aspects (even questions), in order to promote reflection of the answers^17^ ^-^ ^18^. Although it is a unidimensional instrument, factor analysis of the SUS tool shows two factors; usability (questions 1, 2, 3, 5, 6, 7, 8 and 9) and learning (questions 4 and 10). The questions addressed aspects related to use frequency, ease, need for support for handling, functionalities and trust in the app. For the current study, at the end of the assessment two non-mandatory essay questions were added, addressing possible positive and weak points of the app.

Specific instruments were prepared for each group. With regard to the patients, the following information was collected: gender, age, schooling, main diagnosis, transportation means from the residence to the hospital, travel time, place of residence, Internet access frequency, device most used to access the Internet, previous experience with remote health consultations, previous experience with PICCs, safety for outpatient treatment in the use of PICC with the app support, and app handling time. To characterize PICC insertion, the following variables were used: PICC insertion site, number of device lumens, indication of PICC insertion, and PICC use time until data collection. In relation to characterization of the nurses, the gender, age, schooling and time of experience in the care of patients with PICCs variables were used, while for characterization of the ICT professionals, the variables employed were gender, age, schooling and time of professional experience.

### Data sources

The patients’ sociodemographic and clinical information and the PICC insertion data were extracted from the EHRs. The nurses and ICT professionals answered the sociodemographic questionnaire. For usability assessment of the *Meu PICC* app, all the participants answered the SUS instrument. 

### The Meu PICC app for smartphones

**Figure 1:**
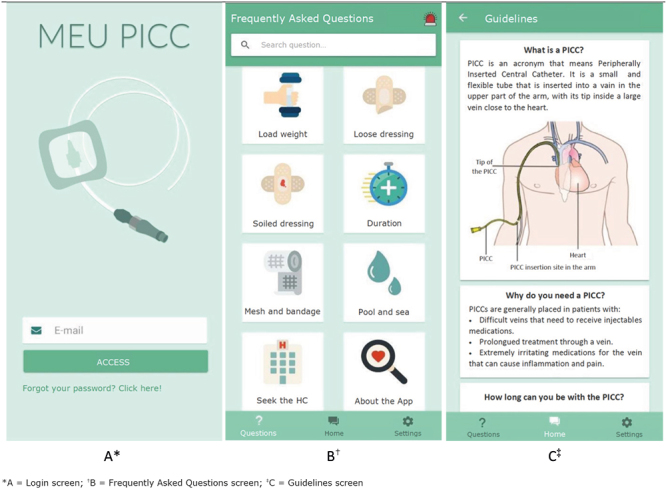
Screens corresponding to Login, Frequently Asked Questions and Guidelines on how to take care of the PICC in the *Meu PICC* app. São Paulo, SP, Brazil, 2020-2021

The *Meu PICC* app was developed with the Scrum methodology, Ionic Angular programming language and Django server, directed to Android and iOS platforms. It has six interactive screens containing the guidelines on how to take care of the PICC, frequently asked questions and the possibility of sending messages and a photograph of the device to the nurse. Figure 1 presents the screens corresponding to Login, Frequently Asked Questions and Guidelines on how to take care of the PICC.

The data related to the patient registration in the app and those shared through the *Chat*, including the photographs sent, are stored in the Firebase platform database, with exclusive access by the principal researcher. Firebase uses the *NoSQL* (Not Only Structured Query Language) storage standard, a non-relational database model that allows for greater speed, flexibility and scalability in data storage when compared to relational databases. In addition to that, it has an encryption system for in-transit and at-rest data, as well as an NBR ISO/IEC 27001:2013 certificate for data safety^19^ and an NBR ISO/IEC 27017 certificate for data safety in cloud services^20^, as stated on its platform^21^. The *Meu PICC* app is registered at the National Institute of Industrial Property (*Instituto Nacional da Propriedade Industrial*, INPI) under process No. BR512021001511-8.

### Data collection procedure

To assess usability, all the participants were approached at the study institution, submitted to the same app tests, as explained below, and answered the SUS instrument. Initially, the study and the app objectives were presented to all three groups of participants. Subsequently, the participants from all groups were asked to use the app through a test user Login (Access) as patients, based on a problem situation related to outpatient PICC use suggested by the researcher herself, in which the participant, regardless of the group, should seek information on how to proceed. Such being the case, the following open question was asked: “Your dressing got wet after the bath and you want to know what should be done. How would you do that?”. For this, the app was used for the period of time that the participant deemed necessary, for up to one hour. Subsequently, in case the participant had not tried to use the Chat function, the researcher asked the second open question: “What would you do to try to contact the nurse in charge?”. After the participant had accessed the Chat, the researcher requested a photograph to be sent through the app itself, followed by the third open question: “The nurse asked you for a photograph to verify the conditions of your catheter. How would you do that?”. 

**Figure 2:**
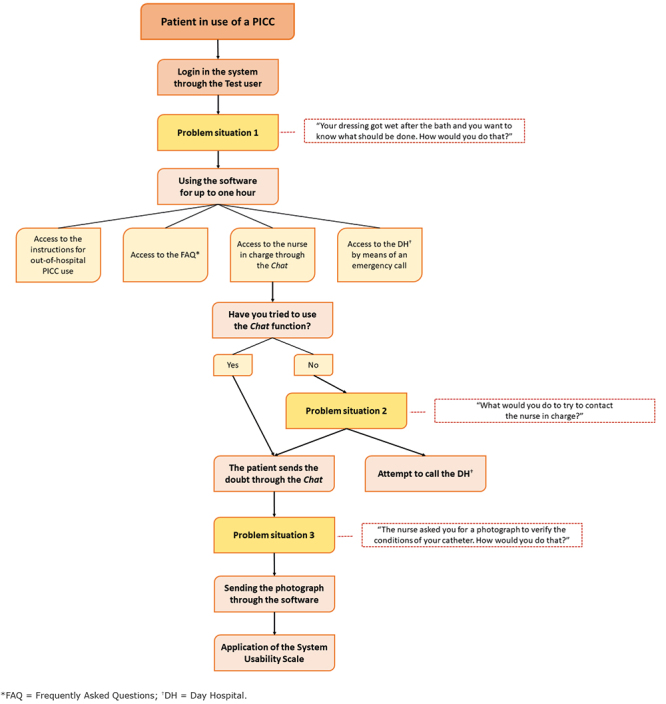
Mental map of the process corresponding to handling and usability assessment of the *Meu PICC* app. São Paulo, SP, Brazil, 2020-2021

After this activity, the SUS scale was applied and there was no interference by the researcher in handling of the app. Figure 2 presents the step-by-step process of this activity.

### Data analysis and treatment

The data were introduced and stored in forms developed in the REDCap (Research Eletronic Data Capture)^22^ system and analyzed in the R software, version 4.0.4.

Central tendency and variability measures were used for the descriptive analysis of the quantitative variables: age, SUS score, app handling time, patient’s travel time to the hospital, PICC permanence time, and time of professional experience of the nurses and ICT professionals. Relative and absolute frequencies were used for the qualitative variables: gender, schooling, main diagnosis, means of transport, place of residence, Internet access frequency, device used to access the Internet, previous experience with Telehealth services, previous experience with PICCs, safety to be discharged with the PICC using the app, PICC insertion site, indications for PICC insertion, and SUS adjectival classification.

Pearson’s correlation coefficient was used to verify the existence of a relationship between the SUS score and the “age”, “PICC use time” and “app handling time” variables. The same correlation coefficient was also used to analyze if there was any relationship between age and app handling time. Kendall’s coefficient was employed to verify if there was any correlation between usability and the “schooling” and “app handling time” variables. The Wilcoxon-Mann-Whitney test was used to verify the existence of a relationship between the usability score and the “gender” and “previous experience with PICCs” variables; as well as the relationship between “gender” and “app handling time”, “PICC use time” and “safety to be discharged with the PICC using the app” variables. Presence or not of an association between the usability score and the subgroups of participants was determined by means of the Kruskal-Wallis test. The existence of a relationship between feeling safe to be discharged with the PICC using the app (dichotomous variable) and age was analyzed by means the Student’s t-test, while for the “gender” and “previous experience with PICCs” variables, the Chi-square association test was used and, for “schooling”, Fisher’s exact test. The statistical significance level adopted was 5.0%. 

To obtain the final SUS score, the following calculations were performed: for the odd answers on the Likert scale (that is, 1, 3 and 5), 1 was subtracted from the score indicated by the user, and 5 was subtracted for the even answers (2 and 4). To calculate the total score, the values obtained from the calculations for even and odd answers were added up and multiplied by 2.5. The overall usability score as *per* the SUS instrument can vary between 0 and 100 points^17^. 

For this study, 70.0% of favorable items was considered as an acceptable parameter, with the sum of the two maximum categories for the odd questions (“I totally agree” and “I agree”) and the sum of the two minimum categories for the even questions (“I totally disagree” and “I disagree”). After calculation of the final score, the app was classified according to the SUS adjectival classification scale, as follows: <20.5 - the worst possible to be imagined; 21-38.5 - deficient; 39-52,5 - average; 53.73.5 - good; 74-85.5 - excellent; 86-100 - the best possible to be imagined^23^ (“imagined” understood as expectation). 

### Ethical aspects

The study met the recommendations set forth in Resolution 466/2016 of the National Council of Ethics in Research. This study represents a stage of the PhD project entitled “Outpatient monitoring of complications through the *Meu PICC* smartphone app in patients using peripherally inserted central catheters treated at a day hospital: A randomized clinical trial”, approved by the Research Ethics Committee of the proposing institution and by the co-participating institution, opinion No. 4,252,374 of September 1^st^, 2020. The participants signed two copies of the Free and Informed Consent Form.

## Results

### Characterization of the patients and use of Information Technologies

Of all 30 patients, 16 (53.3%) were female. The mean age of the patients was 42.1 (SD±13.8) years old, median of 41.5 with a minimum of 19 and a maximum of 63, and there was predominance of patients with incomplete/complete secondary education (n=19; 63.3%). In relation to the main diagnosis, there was predominance of heart conditions, accounting for 21 (70.0%) hospitalizations and followed by pulmonary conditions with nine (30.0%). 

Regarding the means of transport to the hospital, 11 (36.7%) used public transportation, eight (26.7%) private cars, seven (23.3%) app cars, and four (13.3%) municipal vehicles. Travel time to the institution presented a mean of 93.8 (SD±75.2) minutes, median of 85 with a minimum of 10 and a maximum of 360, with 21 (70.0%) patients living in the city of São Paulo and nine (30.0%) in other cities of the state.

Daily and weekly access to the Internet was mentioned by 29 (96.7%) patients and one (3.3%) patient, respectively, preferably through smartphones (n=29; 96.7%) and computers (n=1; 3.3%). In addition to that, four (13.3%) patients reported using some type of Telehealth service. From the point of view of previous experiences with PICCs, 16 (53.3%) patients had already used them in some previous hospitalization and, of these, four (25.0%) had done so in an outpatient context. Of the patients, 24 (80.0%) reported that they felt safe to be discharged with the PICC using the app evaluated, and there was no statistically significant difference when compared to previous PICC use (p=0.280; Chi-square test), schooling (p=0.758; Fisher’s Exact test), gender (0.857; Chi-square test) or the patients’ mean age (p=0.550; Student’s t test). 

Regarding the app handling time, all the participants deemed it necessary to use the app for less than one hour, with a mean of 12 (SD±6.7) minutes and a median of 10, minimum of 5 and maximum of 30. There was no statistically significant difference between the time spent by the patients to evaluate the app and the age (p=0.099), gender (p=0.983) and schooling (p=0.952) variables.

### Characterization of the PICC insertion procedure

The PICCs were preferably inserted in the basilic vein (n=24; 80.0%), followed by the brachial vein in fewer cases (n=5; 16.7%) and, for one procedure, the blood vessel was not recorded in the patient’s EHR. The PICCs had either one or two lumens, with predominance of single-lumen devices (n=20; 66.7%). The indications for PICC insertion were as follows: 18 (60.0%) for the administration of antimicrobials, seven (23.3%) for vasoactive medications and five (16.7%) for other types of IT, such as antivirals, diuretics and anticoagulants.

The mean PICC permanence time was 17.1 (±16.5) days between the day of its insertion and the data collection date, with a median of 14 days, minimum of one and maximum of 86. Greater perception of safety after hospital discharge with PICCs was not observed among the patients with longer PICC use times during hospitalization (p=0.979). 

### Characterization of the nurses and ICT professionals

All the nurses were female, with a mean age of 42,1 (SD±13.8) years old, median of 41.5, minimum of 34 and maximum of 53. Regarding schooling, all nurses had finished a specialization in some specific knowledge area. The mean time of experience in the care of patients with PICCs was 8.9 (SD±3.6) years, with a median of 10, minimum of one year and maximum of 12 years. 

In relation to the ICT professionals, 2 (25.0%) were female and six (75.0%) were male. Their age varied between 31 and 43 years old with a mean of 36.2 (SD±3.8) and a median of 35. Regarding schooling, four (50.0%) had Complete Higher Education, three (37.5%) had some specialization and one (12.5%) was an MSc student. In relation to the experience time in the ICT area, it varied from zero to 17 years, with a mean of 12 (DP±5.2) years and a median of 13.5.

### Usability assessment

The mean SUS score obtained by the patients was 82.7 (SD±16) with a median 85 points, minimum of 40 and maximum of 100; in the case of the nurses, it was 89.2 (SD±9.2), with a median of 91.2 points, minimum of 72 and maximum of 100; while for the ICT professionals the mean score was 85.6 (SD±7.5), with a median of 86.2 points, minimum of 75 and maximum of 95. No statistically significant difference (p=0.561) was observed in the comparison between the groups (Table 1).

**Table 1:** Distribution of the usability assessment score for the app as *per* the System Usability Scale, according to the evaluating group (n=48). São Paulo, SP, Brazil, September 2020 - January 2021

Measurements taken	Score obtained in the System Usability Scale			
	Patients	Nurses	ICT* Professionals	p
**Central Tendency**				
Mean	82.7	89.2	85.6	
Median	85.0	91.2	86.2	
**Variability**				0.561^†^
Standard Deviation	16.0	9.2	7.5	
Minimum	40	72	75	
Maximum	100	100	95	

*ICT = Information and Communication Technology; ^†^Kruskal-Wallis Test

In the comparison of the SUS with the patients’ demographic variables, a statistical difference was only observed for age (p=0.006), with a reduction in usability as age increased, but not for gender (p=0.074) or schooling (p=0.892). There was also a negative correlation between app use time and the score in the usability assessment (p=0.002), that is, the greater the demand for app use time, the lower the usability score.

Considering the total answers obtained from the SUS items, it was observed that, of the total responses provided by the patients (n=300), 7.3% (n=22) evaluated some characteristic of the app in a negative way. As for the total answers provided by the nurses (n=80), this rate corresponded to 3.7% (n=3), whereas this was not observed in relation to the total answers provided by the ICT professionals (n=80). 

It was observed that the percentage of positive answers was below 70.0% on two occasions: in question seven by the patients, which deals with use of the app by most people (66.6% agreement), and in question one by the ICT professionals, which deals with wanting to use the app frequently (62.5% agreement).

As for the SUS adjectival classification, 40.0% of the patients considered the app as the best possible to be imagined and 33.3%, as excellent; 70% of the nurses considered it as the best possible to be imagined and 20.0%, as excellent; and 50.0% of the ICT professionals considered it as the best possible to be and the other 50.0%, as excellent (Table 2).

**Table 2:** Usability assessment of the *Meu PICC* app as *per* the System Usability Scale adjectival classification, according to the evaluating group (n=48). São Paulo, SP, Brazil, September 2020 - January 2021

System Usability Scale adjectival classification	Patients n (%)	Nurses n (%)	ICT* Professionals n (%)
The best possible to be imagined	12 (40.0)	7 (70.0)	4 (50.0)
Excellent	10 (33.3)	2 (20.0)	4 (50.0)
Good	6 (20.0)	1 (10.0)	- (-)
Average	2 (6.7)	- (-)	- (-)
Total	30 (100.0)	10 (100.0)	8 (100.0)

*ICT = Information and Communication Technology

In the essay questions, some participants reported positive points in a generic way or related to practicality and usefulness aspects of the app. Suggestions for improvements related to the diverse information and guidelines or to their location or to the app layout were also presented.

## Discussion

In the current study, it was observed that more than half of the sample had already used PICCs, which is mainly due to the high number of patients with chronic diseases, who may present frequent acute cardiac decompensation, requiring readmissions and new therapy strategies. Non-adherence to the treatment and presence of infections and kidney failure are some of the main reasons for decompensation^24^. Given the above, it was verified that the type of IT most frequently found in the sample was for the administration of antimicrobials and vasoactive drugs. Simultaneous administration of these medications is not uncommon; therefore, INS recommends IT planning, allowing choice of the appropriate device^4^, justifying the use of double-lumen PICCs for one third of the sample.

It is also important to highlight that, as this is a reference institution in cardiopneumology care, it provides assistance to patients from different regions of the country. Thus, it is not uncommon to have patients living in distant locations and, due to the size of the municipality of São Paulo and its metropolitan area, there was a wide variation in the patients’ travel time to the hospital. 

The fact that all the nurses were female corroborates the national profile of this category, in which, according to the Nursing Profile Survey in Brazil conducted in 2013 by the Brazilian Federal Nursing Council (COFEN), 86.2% (n=357,551) of all nurses in the country were female^25^. Also in relation to the same research, there was a slight divergence regarding the mean age, which was higher in the current study when compared to the national mean value, which can be attributed to the profile of nurses with longer experience in the institution.

Regarding the ICT professionals, there was predominance of males and greater variation in the training level, which can be due to their lower mean age when compared to the nurses.

In relation the health education process, it is estimated that between 40.0% and 80.0% of the information provided by the health professionals is immediately forgotten by the patients and, in addition to “health literacy”, different factors can be related, such as: use of technical terminology by the professionals, the patient’s memory deficit caused by age, anxiety and distress caused by the context, perceived importance of the information, and the way in which the information is provided^4^ ^-^ ^5^.

In addition to that, it is important to emphasize that adoption of a new technology requires learning new content and developing new competences, therefore being influenced by cognitive skills. Network access and use of equipment such as computers, tablets and smartphones require visual processing of diverse simultaneous information, rapid recognition of sound signals, and dexterity in the execution of subtle movements such as dragging or clicking on small objects or buttons^26^. 

Over the years, these and other skills undergo changes and may exert an impact on the acceptance and handling of new technologies, which could be observed in this study, where lower scores obtained in the SUS were noticed among older age groups. However, a meta-analysis carried out with 144 studies that evaluated how chronological age is related to acceptance of the use of technologies, observed that the negative effects of age were evident only for technologies considered less useful and with less perceived ease of use^26^. 

Only patients who had access to the Internet and reported mastery in smartphone use were included in this study, as evidenced by the fact that almost all of them mentioned using the device at their homes for daily access to the network. A national survey conducted by *TIC Domicílios* revealed that, in 2008, desktop computers were the main devices used, present in 95.0% of the households with some type of device. In 2019, computer use dropped to 37.0%. This fact is associated with the increase in the use of the network through smartphones: in 2013, these devices were employed by 31.0% of the population and, in 2019, the percentage rose to 78.0%, accounting for approximately 142 million Brazilians^27^.

In recent years, the number of studies involving alternative or complementary use of health-related apps has increased significantly. A multicenter non-inferiority study conducted in Norway observed that the use of smartphone apps and teleconsultations contributed to a reduction in the number of amputations in patients with diabetic foot, proving to be a relevant complementary alternative^28^.

In a clinical trial conducted in the United States of America (USA), which compared complementary monitoring of children receiving home parenteral nutrition through a teleconferencing app, with routine monitoring in the office, a reduction in the rate of Catheter-Related Bloodstream Infection was observed, with 1.0 *per* 1,000 catheters-day in the Intervention Group versus 2.7 in the Control Group^29^.

In the current study, although few participants reported previous experience in Telehealth care, most of them indicated that they would feel safe going home with a PICC using the app. This can be partially attributed to the pandemic caused by the new coronavirus, which required greater use of telehealth services from the entire health system, both public and private, to comply with social distancing and, consequently, greater dissemination of its potentialities^30^. 

The fact that there are no differences in acceptance of the app by patients with and without previous experience in PICC use shows that the app proposed is a useful technology and independent of previous experience. 

The lower SUS scores obtained by the patients in the item that deals with the feasibility of using the app by most people shows that the patients may require greater attention and dedication at the time of discharge regarding training for using the app in the outpatient environment.

When the ICT professionals assigned a lower score to the item of the instrument that points out that the user wants to use the app frequently, it is possible that they interpreted the statement considering use of the app to report complications related to the PICCs. As the *Meu PICC* application is a device to support outpatient PICC use, the fact that it will not be accessed frequently does not disqualify it, if the device remains intact throughout the treatment and the patient is free from complications. In addition, it can mean that the patient education process for discharge was successful.

Some patients required more time to know the app and learn its functions, a fact that was reflected in a lower score in the usability assessment. Despite the positive comments about practicality of the app, there is a need for greater attention in the learning process of those patients who are less familiar with the technology. However, in general, through the mean score obtained in the SUS, it was possible to verify that the app was mostly evaluated as the best possible to be imagined or as excellent in the adjectival classification.

In the literature, studies were found that reported the results related to the use of technologies and their effects on the care process, and not to know-hows, limiting the resources for discussing the results regarding development of the software.

This study presents a positive analysis of the *Meu PICC* app by patients, nurses and ICT professionals, and generates subsidies for the development and evaluation of other apps for education in health, contributing to the advancement of scientific knowledge and corroborating the demands generated by the current scenario imposed by the COVID-19 pandemic, in which it was possible to verify the need for an important increase in health education strategies and interaction between health professionals and patients.

Some limitations were identified in the study, with the unusual situation of the COVID-19 pandemic standing out, which made it difficult to include other participants in the usability assessment of this app, such as availability of patients in outpatient PICC use that could no longer be referred to counter-referral services, and patients’ family members or guardians who were not approached due to the impossibility of visiting and staying in the hospital. The correlation and association analyses had their test power compromised by the sample size, and the literature proved to be scarce with regard to the technological aspects of app development. 

## Conclusion

The usability assessment of the *Meu PICC* app through the System Usability Scale presented a mean score of 82.7 among the patients, 89.2 among the nurses and 85.6 among the Information and Communication Technology professionals, respectively. The participants rated the app as the best possible to be imagined or as excellent, based on the adjectival classification of the scale employed in the usability assessment. 

The results of this assessment generated subsidies for improving the app before proceeding with the comparative intervention study with the app and the routine practice of guiding patients with PICCs in outpatient care.
